# Association between body fat and bone mineral density in Korean adults: a cohort study

**DOI:** 10.1038/s41598-023-44537-1

**Published:** 2023-10-14

**Authors:** Hyunjung Yoon, Eunju Sung, Jae-Heon Kang, Cheol-Hwan Kim, Hocheol Shin, Eunsol Yoo, Minyoung Kim, Mi Yeon Lee, Sujeong Shin

**Affiliations:** 1 Department of Family Medicine, Kangbuk Samsung Hospital, Sungkyunkwan University School of Medicine, 29 Saemunan-Ro, Jongno-Gu, Seoul, 03181 Republic of Korea; 2 Division of Biostatistics, Department of R&D Management, Kangbuk Samsung Hospital, Sungkyunkwan University School of Medicine, Seoul, Republic of Korea

**Keywords:** Diseases, Endocrinology, Health care, Medical research, Risk factors

## Abstract

Although obesity was once considered protective against osteoporosis, various factors influence the relationship between fat and bone mineral density (BMD). To establish the importance of healthy body composition in decelerating declines in BMD, we conducted a study to compare the association between body fat composition and BMD in Korean adults. Using data collected from the Kangbuk Samsung Health Study from 2012 to 2019, this cohort study compared the incidence of decreased BMD among the following four groups: normal BMI and normal adiposity (NBMI-NA), normal BMI and high adiposity (NBMI-HA), overweight, and obesity. Decreased BMD was defined as a Z-score ≤  − 2.0 in premenopausal women and men < 50 years of age or a T-score <  − 1.0 in postmenopausal women and men ≥ 50 years of age. Individuals who were diagnosed with osteoporosis or compression fracture after their second visit were categorized as having decreased BMD. The incidence rate of decreased BMD in the NBMI-NA group was 3.37, and that in the NBMI-HA group was 4.81, which was the highest among all groups. After adjusting for confounding factors, NBMI-HA led to a significantly greater risk of decreased BMD compared to NBMI-NA (HR 1.47; 95% CI 1.09–1.99). Even with a normal BMI, a high BFP was associated with an increased risk of decreased BMD. Therefore, healthy body composition management, not simply BMI, is important in preventing decreased BMD.

## Introduction

The prevalence of osteoporosis is increasing among both women and men, and it is associated with elevated risks of all-cause mortality, mortality from cardiovascular disease, respiratory disease, and cancer^1,2^. Until the age of 30 years, through the remodeling process, reabsorbed bone is replaced with an equal amount of new bone tissue. However, after the age of 30–45 years, an imbalance in bone tissue absorption and formation occurs, and the rate of absorption of bone tissue eventually exceeds the rate of formation^3,4^. Many drugs have been approved for the treatment of osteoporosis to slow the loss of bone density. However, no drug can “cure” osteoporosis. Therefore, an essential goal in reducing the mortality risk is to decelerate the decline in bone mineral density (BMD) prior to the onset of osteoporosis.

Following the start of the coronavirus disease 2019 pandemic, people’s weights increased due to reduced physical activity, overeating, and increased stress^5^. As a result, there has been an increase in the number of individuals attempting to manage their health, with most simply focusing on body mass index (BMI)^6^. In the past, obesity was traditionally considered a protective factor against osteoporosis, resulting in the so-called “obesity paradox^7,8^.” However, recent studies have shown that both multiple factors affect the relationship between bone and adipose tissue, and that fat adversely affects bone tissue^9,10^. Adipose tissue acts as an endocrine organ that promotes bone resorption, inhibits bone formation, and provides mechanical loading to protect against bone loss^11–13^. In previous cross-sectional studies, when weight was corrected to exclude the effects of the physical load of fat, a negative correlation was found between adipose tissue and bone density, and the correlation between fat and bone density varied according to age and sex^14–16^. However, to our knowledge, no cohort study has definitively established the association between high body fat percentage and decreased BMD in individuals, including menopausal women, men or in premenopausal women.

Thus, we conducted a retrospective cohort study to investigate the relationship between body fat and decreased BMD in Korean adults after adjusting for the effects of physical loading on fat. Our study sought to demonstrate that managing body composition for overall health, rather than relying solely on BMI-based health management strategies, is crucial for preventing declines in BMD.

## Method

### Study subjects

A retrospective cohort study was conducted on men and women aged ≥ 18 years who registered in the cohort study database of Kangbuk Samsung Hospital Total Healthcare Center in Seoul and Suwon, Republic of Korea, from January 1, 2012, to December 31, 2019. The study subjects underwent comprehensive health screening examinations at Kangbuk Samsung Hospital Total Healthcare Center. According to the industrial Safety and Health Law in Republic of Korea, all employees of large companies are required to undergo annual or biennial health examinations. Among the study participants, more than 80% were either employees of companies or their family members^17^. Participants who underwent bone density measurement ≥ 2 times by dual-energy X-ray absorptiometry (DXA) bone-density scanning were included in this study. Participants were excluded if they had osteoporosis at the time of their first visit or if they satisfied the World Health Organization (WHO) criteria for osteopenia or osteoporosis based on bone density measurements^18^. Patients were also excluded if information about their demographic characteristics or body measurements was missing or if their body weight was too low (defined by BMI < 18.5 kg/m^2^).

### Anthropometric and biochemical measurements

Based on the Kangbuk Samsung Health Study, the demographic characteristics, body measurements, and biochemical tests of the study participants were investigated. Body fat mass (kg) was measured through bioelectric impedance analysis (Inbody720; Biospace, Seoul, Republic of Korea), and the body fat percentage (BFP) was estimated by dividing body fat mass by body weight^19^. Postmenopausal women were defined as women who responded “yes” to experiencing “no menstruation for > 1 year” in the questionnaire.

### Four categories of BMI and BFP

Participants were classified into four groups according to BMI and BFP values. To investigate the association between body fat and decreased BMD when the BMI was normal, participants with normal BMI were also classified based on a 30% BFP cutoff^20^. Additionally, to confirm the association between obesity and decreased BMD, study participants with high BMIs were divided into overweight and obesity groups. The WHO Asia–Pacific Centre and the Korean Society of Obesity define patients with a BMI of 23–24.9 kg/m^2^ as overweight and those with a BMI ≥ 25 kg/m^2^ as obese. The diagnostic criteria used in this study for assigning participants into the overweight and obesity groups by BMI were based on these criteria^21,22^, and the final four study groups were as follows: (1) normal BMI and normal adiposity (NBMI-NA), BMI of 18.5–22.9 kg/m^2^ and BFP < 30%; (2) normal BMI and high adiposity (NBMI-HA), BMI of 18.5–22.9 kg/m^2^ and BFP ≥ 30%; (3) overweight, BMI of 23–24.9 kg/m^2^; and (4) obesity: BMI ≥ 25 kg/m^2^.

### Measurement of BMD

BMD was measured by DXA (Prodigy [GE Healthcare, Madison, WI, USA] or HOLOGIC QDR 4500W [Hologic Inc., Bedford, MA, USA]). In premenopausal women and men < 50 years of age, a Z-score was obtained by comparing each patient’s BMD with the average BMD of the same age group. In postmenopausal women and men ≥ 50 years of age, the T-score was obtained by comparing each patient’s BMD with the BMD of the young adult group.

### Definition of decreased BMD

A decreased BMD was recorded when ≥ 1 of the following three conditions was satisfied: (1) a total lumbar or femur neck or total femur Z-score ≤  − 2.0 in premenopausal women or men < 50 years of age during the second and subsequent BMD measurement through DXA within the study period; (2) a total lumbar or femur neck or total femur T-score <  − 1.0 in postmenopausal women or men > 50 years of age during the second and subsequent BMD measurement through DXA, based on the WHO criteria of osteopenia and osteoporosis^18^; and (3) diagnosed with osteoporosis, had taken medication for osteoporosis, or had a compression fracture as recorded during the second and subsequent questionnaires within the study period.

### Statistical analysis

Statistical analysis was conducted according to the four categories of BMI and BFP. Continuous variables were described with mean and standard deviation values, and mean comparisons among the four groups were analyzed by one-way analysis of variance. If the distribution was not normal, the median (interquartile range) value was tested with the Kruskal–Wallis H test. Categorical variables were described by frequency and ratio, and Pearson’s chi-square test was used to test the difference in ratios between the four groups. To confirm the incidence of decreased BMD in each group, the total number of person-years was calculated by summing the observation periods of the study subjects (Table 2). The observation period was defined as the time from occurrence of decreased BMD relative to the first DXA measurement. The incidence rate per 1000 person-years was calculated by dividing the number of people with decreased bone density by the total number of person-years and multiplying by 1000. Hazard ratios (HRs) and 95% confidence intervals (CIs) for decreased BMD in the four groups were obtained using a Cox proportional hazards model with adjustments for confounders (Table 3). Statistical analyses were performed using STATA version 17.1 (StataCorp LLC, College Station, TX, USA).

### Ethical approval

The present study did not include any animal studies. All procedures involved in this study of human participants were in accordance with the ethical standards of the institutional research committee and with the 1964 Helsinki declaration and its later amendments or comparable ethical standards. This study was conducted with approval from the Institutional Review Board (IRB) of Kangbuk Samsung Hospital (IRB no. 2022-10-055), which waived the requirement for informed consent as pre-existing de-identified data obtained from the Kangbuk Samsung Cohort Study was used for this study.

## Results

### Comparison of baseline characteristics

**Figure 1:**
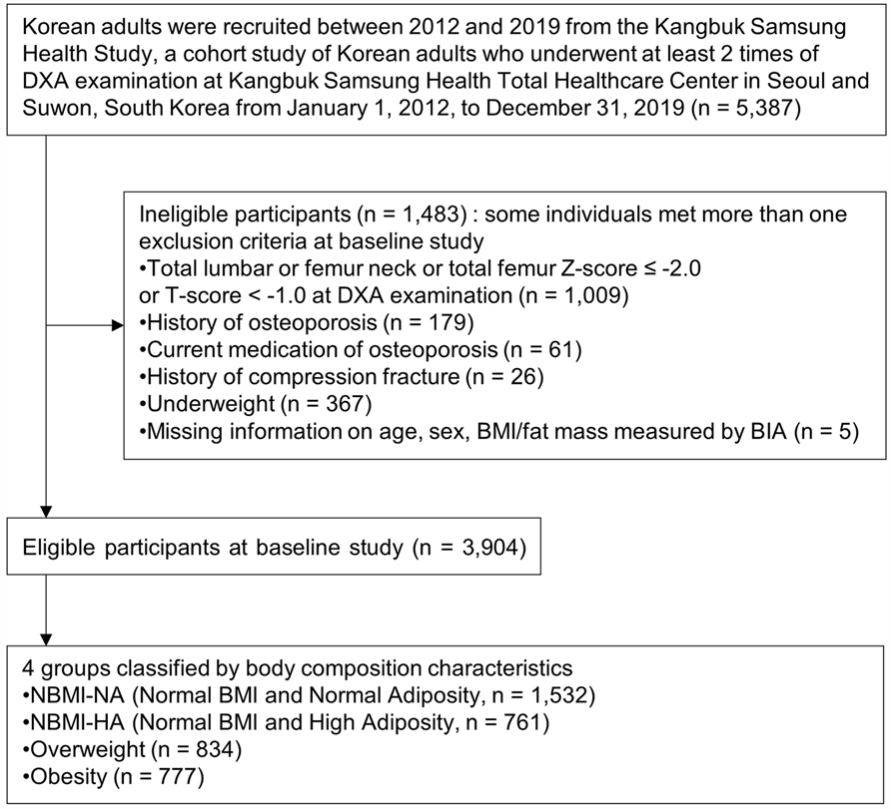
Flow chart of study participants.

There were 3904 eligible participants for this study, including 3521 women and 383 men (Fig. 1). As shown in Table 1, the average age of the NBMI-NA and NBMI-HA groups was approximately 46.5 years, the average age of the overweight group was 49.4 years, and the average age of the obese group was 50.1 years. The average BMIs were 20.6 kg/m^2^ in the NBMI-NA group, 21.6 kg/m^2^ in the NBMI-HA group, 23.89 kg/m^2^ in the overweight group, and 27.45 kg/m^2^ in the obesity group. Notably, the mean BFP values of the NBMI-HA and overweight groups were similar (25.24% in the NBMI-NA group, 32.89% in the NBMI-HA group, 32.04% in the overweight group, and 36.28% in the obesity group). eTable 1 in the Supplementary section lists the characteristics of female participants, which were similar to those of all study participants.

**Table 1:** Comparison of baseline characteristics according to the categories of body mass index and body fat percentage.

Baseline characteristics	Total (n = 3904)			
	NBMI-NA (n = 1532, 39.24%)	NBMI-HA (n = 761, 19.49%)	Overweight (n = 834, 21.36%)	Obese (n = 777, 19.9%)
Age (y)	46.5 ± 6.95	46.48 ± 5.76	49.39 ± 7.92	50.13 ± 8.07
Height (cm)	161.04 ± 5.48	159.19 ± 5	160.15 ± 6.19	160.35 ± 6.31
Body weight (kg)	53.5 ± 4.85	54.77 ± 4.07	61.38 ± 5.05	70.68 ± 8.32
Waist circumference (cm)	72.34 ± 4.93	75.29 ± 4.26	80.85 ± 4.78	89.07 ± 6.8
BMI (kg/m^2^)	20.6 ± 1.15	21.6 ± 0.98	23.89 ± 0.57	27.45 ± 2.51
BFP (%)	25.24 ± 3.52	32.89 ± 2.13	32.04 ± 4.94	36.28 ± 5.66
History of rheumatoid arthritis	17 (1.11)	8 (1.05)	11 (1.32)	10 (1.29)
Subjects who have taken oral steroids for ≥ 3 months (%)^3^	5 (0.33)	0 (0)	2 (0.24)	0 (0)
Subjects who drink alcohol 10 g/day (%)	211 (13.77)	65 (8.54)	126 (15.11)	141 (18.15)
Subjects who have smoked (%)	250 (16.32)	76 (9.99)	157 (18.82)	169 (21.75)
Subjects who do regular vigorous physical activities ≥ 3 times/week (%)	260 (16.97)	86 (11.3)	137 (16.43)	126 (16.22)

Values are presented as mean + /- standard deviation or median or number (%).

Continuous variables were described as average values and standard deviation, and average comparison between four groups classified by body composition was analyzed using a one-way ANOVA test. In cases of a violation of the normal distribution, it was described as the median (interquartile rage) and tested through a Kruskal–Wallis H test. Categorical variables were described as frequency and ratio, and the Pearson’s chi-squared test was used to test the ratio difference between the four groups.

*NBMI-NA* normal BMI and normal adiposity, *NBMI-HA* normal BMI and high adiposity, *BMI* body mass index, *BFP* body fat percentage, *BMD* bone mineral density.

### Incidence of decreased BMD

Table 2 lists the incidence of decreased BMD in all study participants and female participants only. Regardless of sex, the total number of person-years of all participants was approximately 9527, and the median follow-up period was 2.03 years. During the observation period of a total of 9527 person-years, 336 people presented with reductions in BMD, and the incidence rate per 1000 person-years was 3.53. When comparing the incidence per 1000 person-years of decreased BMD across the four groups, the incidence rate in the NBMI-NA group was 3.37 and that in the NBMI-HA group was 4.81, which was the highest among all groups. When looking at the incidence of the decrease in bone density in female participants only, during the observation period of a total of 8322 person-years, decreased BMD was observed in 305 women, and the incidence rate per 1000 person-years was 3.66. In female participants, the incidence rate of the NBMI-NA group was 3.28 and that of the NBMI-HA group was 4.81, which was also the highest among all groups, just like among all study participants.

**Table 2:** Incidence of decreased bone mineral density according to the categories of body mass index and body fat percentage.

	Person-years	Number of incidents	Incidence rate per 1000 person-years
Total			
All	9526.56	336	3.53 (3.17–3.93)
NBMI-NA	3678.29	124	3.37 (2.83–4.02)
NBMI-HA	1808.79	87	4.81 (3.9–5.93)
Overweight	2060.25	66	3.2 (2.52–4.08)
Obesity	1979.24	59	2.98 (2.31–3.85)
Women			
All	8322.26	305	3.66 (3.28–4.1)
NBMI-NA	3265.85	107	3.28 (2.71–3.96)
NBMI-HA	1808.79	87	4.81 (3.9–5.93)
Overweight	1670.77	58	3.47 (2.68–4.49)
Obesity	1576.85	53	3.36 (2.57–4.4)

*NBMI-NA* normal BMI and normal adiposity, *NBMI-HA* normal BMI and high adiposity.

Table 3 shows the risk ratio of decreased BMD incidence for the NBMI-HA, overweight, and obesity groups in comparison to the incidence in the NBMI-NA group. For model 1, we adjusted for age and sex in males and additionally adjusted for menopausal status in female participants. In model 2, we additionally adjusted for BMI to adjust for the effects of physical load. In model 3, we adjusted for a history of rheumatoid arthritis, oral steroid usage, smoking, drinking, and physical activity^23^. Among all study participants, the NBMI-HA group showed a significantly greater risk of decreased BMD compared to the NBMI-NA group (in model 2: HR 1.43; 95% CI 1.07–1.93; in model 3: HR 1.47; 95% CI 1.09–1.99). The risk ratio of the overweight and obesity groups was greater than that of the NBMI-NA group (although not statistically significant) in model 2 and model 3. In only female participants, the NBMI-HA group showed a significantly higher risk of decreased BMD compared to the NBMI-NA group, which was in accordance with that of all study participants (in model 2: HR 1.52; 95% CI 1.11–2.07; in model 3: HR 1.56; 95% CI 1.14–2.13). In models 2 and 3, the obesity group showed a significantly higher risk of decreased BMD compared to the NBMI-NA group when considering only female participants.

**Table 3:** Decreased bone mineral density according to the categories of body mass index and body fat percentage.

	Unadjusted	Model 1	Model 2	Model 3
Total				
NBMI-NA	1 (reference)	1 (reference)	1 (reference)	1 (reference)
NBMI-HA	1.47 (1.12–1.94)*	1.21 (0.92–1.6)	1.43 (1.07–1.93)*	1.47 (1.09–1.99)*
Overweight	0.91 (0.68–1.23)	0.75 (0.55–1.02)	1.24 (0.8–1.91)	1.28 (0.83–1.97)
Obesity	0.85 (0.62–1.16)	0.68 (0.5–0.94)	1.87 (0.96–3.63)	1.89 (0.97–3.69)
Women				
NBMI-NA	1 (reference)	1 (reference)	1 (reference)	1 (reference)
NBMI-HA	1.45 (1.09–1.92)*	1.26 (0.94–1.67)	1.52 (1.11–2.07)*	1.56 (1.14–2.13)*
Overweight	1.02 (0.74–1.4)	0.8 (0.58–1.11)	1.36 (0.85–2.18)	1.41 (0.88–2.27)
Obesity	1 (0.72–1.39)	0.74 (0.53–1.04)	2.16 (1.05–4.44)*	2.21 (1.07–4.55)*

Values are presented as adjusted hazard ratio (95% confidence interval), *p < 0.05.

Model 1: adjusted for age, sex, menopause; Model 2: adjusted for age, sex, menopause, BMI; Model 3: adjusted for model 2 + history of rheumatoid arthritis, medication of steroid, smoking, alcohol consumption, physical activity.

*NBMI-NA* normal BMI and normal adiposity, *NBMI-HA* normal BMI and high adiposity.

## Discussion

To the best of our knowledge, this is the first retrospective cohort study confirming the association between body fat and decreased BMD in Korean adults, including not only menopausal women, but also men and premenopausal women. In this cohort study, the incidence of decreased BMD was highest in the NBMI-HA group, with greater risk of decreased BMD compared to in the NBMI-NA group, regardless of the adjustments made. This suggests that, even if a person’s BMI is normal, the risk of decreased BMD increases if the BFP is high (BFP ≥ 30%).

Our results reinforce the findings from a Korean population study that showed a negative correlation between fat mass and BMD in women^16^. Moreover, previous studies have demonstrated that fat mass has a negative effect on bone mass in contrast with the positive effect of weight-bearing itself, and this aligns with the findings of this cohort study^14,15^. Therefore, we suggest that a personalized approach considering body fat is necessary for confirming bone health.

In a previous cross-sectional study using data from the Korea National Health and Nutrition Examination Survey from 2008 to 2011, fat mass positively affected the BMD of women and older men with normal BMIs^24^, which contradicts the results of this cohort study. However, our cohort study has the following strengths compared to the cross-sectional study. First, unlike the cross-sectional study that did not adjust for confounding factors, such as BMI, rheumatoid arthritis, and steroid use, this cohort study included these variables. Second, by confirming a positive correlation between BFP and decreased BMD, including in younger adults (premenopausal women and men < 50 years of age), this cohort study highlights the need for a healthy body composition management for bone health from a young age. With a previous study showing how body fat distribution plays an important role in determining bone density in young adults^25^, future cohort studies that consider body fat distribution will also be necessary.

The “obesity paradox” has been a source of confusion in metabolic studies^7,8^, but recent studies have shown that there are various factors affecting both fat and bone tissue, and the connection between them is not clear^9–13^. There is a variety of factors involved in the association between adipose tissue and bone tissue, including mechanical factors, metabolic factors, and hormones^9^. Mechanical association would be best explained by the increased physical loading due to adipose tissue^26^. However, physical loading alone is not sufficient to fully explain the interaction between adipose tissue and bone density^27^. Various hormones and cytokines are known to influence the interaction between adipose tissue and bone tissue. Aromatase, which synthesizes the estrogen that promotes bone formation and reduces bone resorption, is secreted not only in the gonads, but also in adipose tissue^28^. Although adipose tissue is thought to contribute to bone health through this mechanism, there are other factors involved as well. Leptin, which is mainly secreted from adipocytes, has been reported to have both positive^29^ and negative effects^30,31^ on bone formation, making it controversial. Adiposity is associated with the increase in inflammatory factors like C-reactive protein, tumor necrosis factor–α, and interleukin-6, which inhibit adiponectin that increases bone density^32^. Additionally, tumor necrosis factor–α and interleukin-6 promote osteoclastic resorption^33,34^. Other factors, such as vitamin D level and differences in adipose tissue distribution (visceral fat, subcutaneous fat) are involved in the association between fat and bone tissue, but the impact of these factors is still controversial^35,36^. Although it was difficult to confirm the effects of these various factors in this cohort study, this study still provided evidence that body fat and bone health are negatively correlated.

This study has several limitations. First, there are several factors that can affect the measurements obtained from a bioelectric impedance analysis (Inbody720; Biospace, Seoul, Republic of Korea), including hydration status, recent food and drink consumption, physical activity level, and menstrual cycle. However, all participants at the Kangbuk Samsung Hospital Total Healthcare Center underwent bioelectric impedance analysis measurements following the standard protocol, which included an 8-h fasting period and urination within 30 min prior to measurement to enhance the precision of the obtained results. Second, although the observed association has been adjusted extensively for risk factors of decreased BMD, other unmeasured confounders such as vitamin D or vitamin A status may contribute to the association^3,35^. Third, it is difficult to generalize the findings to the entire adult population of Republic of Korea. Majority of the participants were current employees, or family members of large companies. Furthermore, only a small number of men underwent DXA scanning, resulting in a significantly lower number of male participants included in this study. However, despite the small number of male participants, the results showed an association between reduced risk of increased BMD with high body fat percentage within the normal BMI range. Fourth, while the NBMI-HA group showed a greater prevalence of decreased BMD compared to the obesity group (as seen in Table 2), the risk ratio of decreased BMD for the NBMI-HA group was lower than that of the obesity group when considering only female participants. Given the diverse factors influencing the relationship between fat and bones^37^, this warrants further investigation.

In conclusion, the incidence of decreased bone density was highest in the NBMI-HA group, and the risk of decreased bone density was statistically significantly higher in the NBMI-HA group than in the NBMI-NA group, regardless of the adjustments made. This suggests that, even if the recorded BMI was normal, a high BFP is associated with an increased risk of decreased bone density. At present, there has been an increase in the number of individuals attempting to manage their health, with most simply focusing on BMI^6^. However, the results of this study confirm that focusing on a healthy body composition, not simply BMI, is more important in preventing decreased BMD. This could lead to significant implications for the development of effective preventive strategies for osteoporosis.

### Supplementary Information

Supplementary Table 1.

## Data Availability

The datasets used and/or analyzed during the current study are not publicly available outside the hospital due to institutional review board limitations, which prevent widespread dissemination of the data. However, the datasets are available from the corresponding author on reasonable request and with permission of the institutional review board.
