# Supplementary material for: Association between body fat and bone mineral density in Korean adults: a cohort study
Source: Sci Rep. 2023 Oct 14;13:17462. doi: 10.1038/s41598-023-44537-1 (PMC10576818; doi:10.1038/s41598-023-44537-1)
Supplement: Supplementary file 1 — Supplementary Table 1. [file 41598_2023_44537_MOESM1_ESM.docx]

**Supplementary**

**eTable1.** Comparison of baseline characteristics according to the categories of BMI and BFP in women study participants

| Baseline characteristics | Total (n = 3,521) | | | |
| --- | --- | --- | --- | --- |
|  | NBMI-NA  (n = 1401, 39.79%) | NBMI-HA  (n = 761, 21.61%) | Overweight  (n = 711, 20.19%) | Obese  (n = 648, 18.4%) |
| Age (y) | 45.35 ± 5.36 | 46.48 ± 5.76 | 47.51 ± 6.23 | 48.21 ± 6.61 |
| Height (cm) | 160.32 ± 4.87 | 159.19 ± 5 | 158.66 ± 5 | 158.67 ± 4.92 |
| Body weight (kg) | 52.77 ± 4.11 | 54.77 ± 4.07 | 60.13 ± 3.96 | 69.36 ± 7.85 |
| Waist circumference | 71.66 ± 4.32 | 75.29 ± 4.26 | 79.96 ± 4.29 | 88.19 ± 6.78 |
| BMI (kg/m^2^) | 20.52 ± 1.11 | 21.6 ± 0.98 | 23.87 ± 0.57 | 27.52 ± 2.62 |
| BFP (%) | 25.79 ± 2.95 | 32.89 ± 2.13 | 33.53 ± 3.37 | 38 ± 4.08 |
| History of rheumatoid arthritis | 15 (1.07) | 8 (1.05) | 9 (1.27) | 8 (1.23) |
| Subjects who have taken oral steroids for ≥ 3 months (%)^3^ | 5 (0.36) | 0 (0) | 2 (0.28) | 0 (0) |
| Subjects who drink alcohol 10 g/day (%) | 144 (10.28) | 65 (8.54) | 62 (8.72) | 68 (10.49) |
| Subjects who have ever smoked (%) | 155 (11.06) | 76 (9.99) | 66 (9.28) | 73 (11.27) |
| Subjects who do regular vigorous physical activities ≥ 3 times/week (%) | 237 (16.92) | 86 (11.3) | 110 (15.47) | 94 (14.51) |

Values are presented as mean +/- standard deviation or median or number (%)

Continuous variables were described as average values and standard deviation, and average comparison between four groups classified by body composition was analyzed using a one-way ANOVA test. In case of a violation of the normal distribution, it was described as the median (interquartile rage) and tested through a Kruskal–Wallis H test. Categorical variables were described as frequency and ratio, and the Pearson’s chi-squared test was used to test the ratio difference between the four groups.

*NBMI-NA* normal BMI and normal adiposity, *NBMI-HA* normal BMI and high adiposity, *BMI* body mass index, *BFP* body fat percentage, *BMD* bone mineral density
